# Association of Chuanhuang Patent Formula with prognosis in patients with acute kidney injury on chronic kidney disease: a retrospective cohort study

**DOI:** 10.1080/0886022X.2026.2713828

**Published:** 2026-08-13

**Authors:** Yifan Zhang, Zhong Wang, Qiuhan Wang, Zhiyong Song, Xuezhong Gong

**Affiliations:** Department of Nephrology, Shanghai Municipal Hospital of Traditional Chinese Medicine, Shanghai University of Traditional Chinese Medicine, Shanghai, China

**Keywords:** Acute kidney injury, Chuanhuang Patent Formula, chronic kidney disease, retrospective study, traditional Chinese medicine

## Abstract

This study evaluated the association of Chuanhuang Patent Formula (CHPF) with short-term renal function parameters and long-term prognosis in patients with acute kidney injury (AKI) on chronic kidney disease (CKD) (A on C) and explored potential subgroups that might derive greater benefit. This retrospective cohort included 205 patients with A on C admitted between January 2016 and October 2025. After 1:1 propensity score matching, 81 patients were included in each group. The primary composite outcome was progression to CKD stage 5, maintenance renal replacement therapy initiation, or all-cause mortality, while secondary outcomes evaluated renal function parameters at 2 and 4 weeks. Renal function parameters were more favorable in the CHPF group at 2 and 4 weeks after treatment initiation. Long-term survival analysis showed that CHPF treatment was associated with a lower risk of the primary outcome (hazard ratio [HR] = 0.56; 95% confidence interval [CI]: 0.32–0.98; *p* = 0.041). Notably, hypertension-stratified subgroup analysis showed that the association between CHPF treatment and outcome-free survival appeared more evident in patients with comorbid hypertension (*p* = 0.045), whereas no significant difference was observed in patients without hypertension (*p* = 0.26). In conclusion, CHPF could be a promising adjunctive therapeutic strategy for patients with A on C and was associated with more favorable 2- and 4-week renal function parameters and long-term outcome-free survival. Patients with comorbid hypertension might represent a potential subgroup that could derive greater benefit from CHPF treatment; however, this exploratory finding should be interpreted cautiously and validated in prospective studies.

## Lay summary

Preexisting chronic kidney disease (CKD) is an important risk factor for acute kidney injury (AKI), and an AKI episode could accelerate progression to end-stage kidney disease. This retrospective cohort study evaluated the association between Chuanhuang Patent Formula (CHPF), a traditional Chinese medicine formula, and renal function and long-term outcomes in patients with AKI on CKD. After propensity score matching, patients who received CHPF in addition to standard supportive care had more favorable renal function parameters at 2 and 4 weeks and a lower risk of progression to CKD stage 5, initiation of maintenance renal replacement therapy, or all-cause mortality than those who received standard supportive care alone. These findings suggest that CHPF could be a potential adjunctive therapeutic strategy for this high-risk population, although prospective studies are needed for further validation.

## Key messages

**What is known:** Acute kidney injury (AKI) on chronic kidney disease (CKD) is associated with accelerated renal function decline and poor long-term prognosis, while effective treatment options remain limited.

**This study adds:** In this retrospective cohort study with propensity score matching, CHPF treatment was associated with more favorable 2- and 4-week renal function parameters and a lower risk of the long-term primary composite outcome.

**Potential impact:** CHPF could represent a promising adjunctive therapeutic strategy for patients with AKI on CKD, and patients with comorbid hypertension warrant further prospective investigation.

## GRAPHICAL ABSTRACT

**Figure:**
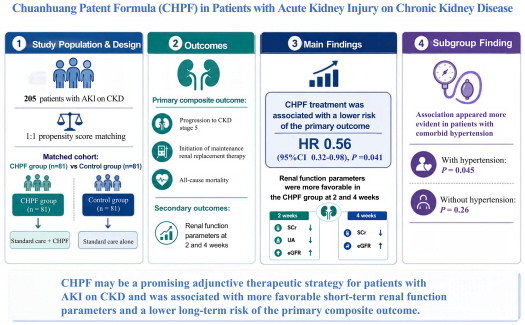

## 1. Introduction

Acute kidney injury (AKI) is defined as a rapid decline in renal function, typically characterized by elevated serum creatinine levels and decreased urine output [1]. Approximately, 10–15% of hospitalized patients develop AKI; notably, the incidence exceeds 50% among patients in the intensive care unit, a condition closely associated with increased in-hospital mortality [2]. The etiology of AKI is complex, involving prerenal, intrinsic renal, and postrenal factors, and its clinical impact could be further aggravated in patients with preexisting chronic kidney disease (CKD) [3,4]. Multiple risk factors, including advanced age, CKD, diabetes, and hypertension, could contribute to pathological processes such as renal ischemia, inflammation, oxidative stress, maladaptive repair, and renal fibrosis, ultimately resulting in renal impairment and CKD progression [5–9]. Although the Kidney Disease: Improving Global Outcomes (KDIGO) guidelines recommend renal replacement therapy (RRT) as the final supportive measure for severe AKI (stage 3), specific pharmacological interventions to arrest disease progression during the early-to-moderate stages (stages 1 and 2) remain lacking [10].

CKD refers to persistent structural or functional abnormalities of the kidney lasting more than three months [11]. The onset of CKD is insidious, often with a prolonged latency period. Once patients progress to end-stage renal disease (ESRD), survival relies on RRT, imposing a massive economic burden on individuals, families, and society while severely threatening patient survival. Globally, CKD affects 674 million people, with an age-standardized prevalence of 8.006% [12]. There is a profound correlation between AKI and CKD. Preexisting CKD is a confirmed major risk factor for AKI; compared to the non-CKD population, patients with CKD face up to a 10-fold increased risk of developing AKI [13]. This high susceptibility suggests that CKD and AKI are not independent entities but interconnected clinical syndromes. Furthermore, AKI is a distinct risk factor for CKD progression. Studies indicate that patients with a history of AKI have a 2.67-fold increased risk of incident CKD or progression of existing CKD, and a 4.81-fold increased risk of progressing to ESRD compared to those without AKI [14]. AKI has become a critical ‘catalyst’ accelerating the progression of CKD patients toward ESRD; therefore, identifying effective strategies to interrupt this malignant process is urgent.

In the face of the intractable clinical challenge of AKI on CKD (A on C), traditional Chinese medicine (TCM) has been increasingly investigated as a complementary preventive and therapeutic strategy, leveraging its unique theoretical system, extensive clinical practice, and multi-component, multi-target characteristics [15,16]. Chinese herbal medicines and their active ingredients have shown potential renoprotective effects, primarily through mechanisms involving the regulation of oxidative stress, inhibition of inflammation, attenuation of renal fibrosis, and modulation of regulated cell death. These multi-component and multi-target regulatory characteristics provide a theoretical basis for their potential role in improving renal outcomes [17–22]. Chuanhuang Fang (CHF), a Chinese herbal formula developed by Professor Xuezhong Gong of the Shanghai Municipal Hospital of Traditional Chinese Medicine, is currently used in multiple hospitals in China for the treatment of A on C [23,24]. Our previous studies suggested that CHF may exert renoprotective effects by reducing serum creatinine (SCr), uric acid (UA), blood urea nitrogen (BUN), and AKI-related biomarkers such as urinary neutrophil gelatinase-associated lipocalin, serum human epididymis protein 4, and N-terminal pro-brain natriuretic peptide levels [25]. Additionally, our previous randomized controlled trials in patients with A on C suggested that CHF combined with prostaglandin E1 attenuates inflammatory responses by lowering levels of NLR family pyrin domain containing 3 and inflammatory factors such as interleukin-6, improves TCM syndrome scores, and promotes the recovery of renal function [26,27].

Based on our previous studies of CHF, our research group subsequently developed and patented Chuanhuang Patent Formula (CHPF). This study employed a retrospective analysis of real-world data combined with propensity score matching (PSM) to evaluate the association of a CHPF-based herbal medicine regimen with short-term renal function parameters and long-term prognosis in patients with A on C.

## 2. Methods

### 2.1. Study design and population

This single-center, retrospective cohort study included 205 patients with A on C admitted to the Shanghai Municipal Hospital of Traditional Chinese Medicine between January 2016 and October 2025. The study protocol was approved by the Medical Ethics Committee of the hospital (No. 2026SHL-KY-39-01) and was conducted in accordance with the Declaration of Helsinki. Due to the retrospective nature of the study and the use of de-identified data, the requirement for informed consent was waived.

AKI was defined according to the 2012 KDIGO Clinical Practice Guideline for Acute Kidney Injury [10]: stage 1 was defined as an increase in SCr by 1.5–1.9 times baseline, or ≥0.3 mg/dL (≥26.5 µmol/L) increase, or urine output <0.5 mL/kg/h for 6–12 h; stage 2 was defined as SCr 2.0–2.9 times baseline, or urine output <0.5 mL/kg/h for ≥12 h; stage 3 was defined as SCr 3.0 times baseline, or SCr ≥ 4.0 mg/dL (≥353.6 µmol/L), or initiation of RRT, or in patients <18 years, a decrease in estimated glomerular filtration rate (eGFR) to <35 mL/min/1.73 m^2^, or urine output <0.3 mL/kg/h for ≥24 h, or anuria for ≥12 h.

CKD was defined as structural or functional abnormalities of the kidney present for >3 months, in accordance with KDIGO guidelines [28]. For patients with an eGFR ≥60 mL/min/1.73 m^2^, the diagnosis of CKD required additional evidence of kidney damage, such as persistent proteinuria/albuminuria or structural abnormalities of the kidney. CKD staging was classified as follows: stage 1, eGFR ≥ 90 mL/min/1.73 m^2^; stage 2, eGFR 60–89 mL/min/1.73 m^2^; stage 3, eGFR 30–59 mL/min/1.73 m^2^; stage 4, eGFR 15–29 mL/min/1.73 m^2^; and stage 5, eGFR < 15 mL/min/1.73 m^2^. The 2021 Chronic Kidney Disease Epidemiology Collaboration creatinine equation was used to calculate eGFR.

The inclusion criteria were as follows: (1) age ≥ 18 years; (2) met the diagnostic criteria for CKD stages 2–4 and AKI stages 1–2; (3) received Western medicine and/or CHPF for ≥2 weeks after the onset of AKI; and (4) available medical records and follow-up data. The exclusion criteria were as follows: (1) pregnancy or lactation; (2) severe dysfunction of other organs; and (3) known allergy or physiological intolerance to the study medications.

### 2.2. Interventions

All patients received standard supportive care, including a low-salt, high-quality low-protein diet (protein intake 0.6–0.8 g/kg/day), correction of water, electrolyte, and acid–base imbalances, blood pressure control, and management of anemia and CKD-mineral and bone disorder. In addition to standard care, patients in the CHPF group were administered CHPF orally twice daily for at least two weeks. CHPF consists of Chuanxiong (Chuanxiong Rhizoma), Zhidahuang (Rhei Radix et Rhizoma Praeparata), Dangshen (Codonopsis Radix), Zisu (Perillae Folium), Chenpi (Citri Reticulatae Pericarpium), and Jinchanhua (Paecilomyces cicadae). The formulation has been granted a Chinese patent (Chinese Patent No. ZL201910542982.X). All herbal materials were purchased from the Pharmacy of Shanghai Municipal Hospital of Traditional Chinese Medicine and were prepared as a decoction by the hospital pharmacy. In contrast, patients in the control group received standard supportive care alone during the efficacy evaluation period, without the administration of any Chinese herbal preparations.

### 2.3. Data collection

Demographic and clinical data were extracted from electronic medical records by trained researchers using a standardized data collection form. The collected data included age, sex, body mass index, systolic blood pressure, diastolic blood pressure, comorbidities (diabetes mellitus, hypertension, and coronary heart disease), laboratory indicators (albumin (ALB), SCr, BUN, UA, eGFR, and urinary albumin-to-creatinine ratio (ACR)), AKI etiology, and use of renin–angiotensin–aldosterone system (RAAS) inhibitors and statins. AKI etiology was classified according to the documented causes or precipitating factors in the electronic medical records as nephrotoxic agent, infection, other causes, or unclear. Cases without a clearly documented AKI etiology were categorized as unclear. All data were double-checked by a second researcher after entry, and any discrepancies were resolved through discussion with the principal investigator.

### 2.4. Outcomes

The primary outcome was a composite endpoint consisting of: (1) progression to CKD stage 5, defined as sustained eGFR < 15 mL/min/1.73 m^2^ for ≥4 weeks; (2) initiation of maintenance RRT; and (3) all-cause mortality. Secondary outcomes included the levels of SCr, BUN, UA, and eGFR recorded at 2 and 4 weeks after treatment initiation. The definition of the primary composite outcome was based on clinically meaningful long-term kidney outcomes and the concept of major adverse kidney events, which commonly include death, dialysis dependence, and persistent renal dysfunction [29,30].

### 2.5. Statistical analysis

Continuous variables are presented as mean ± standard deviation (mean ± SD) or median (interquartile range, IQR) based on the data distribution. Categorical variables are presented as counts (percentages). For group comparisons, continuous variables were analyzed using the independent samples *t*-test or Mann–Whitney’s *U*-test, and categorical variables were analyzed using the chi-square (*χ*^2^) test. For intragroup comparisons, the paired *t*-test or Wilcoxon’s signed-rank test was employed. Among the baseline covariates, only ACR had missing data, with a missing proportion of 10.7%; missing ACR values were handled using multiple imputation. The Cox proportional hazards regression model was used to evaluate risk factors for the primary outcome, and multivariate models were constructed to adjust for potential confounders. Three Cox regression models were constructed to assess the robustness of the association between CHPF treatment and the primary composite outcome. Model 1 was unadjusted; model 2 was adjusted for age and sex; and model 3 was further adjusted for clinically relevant baseline variables that could influence prognosis or treatment allocation, including age, sex, body mass index, systolic blood pressure, diastolic blood pressure, diabetes mellitus, hypertension, coronary heart disease, RAAS inhibitors use, statin use, AKI stage, AKI etiology, ALB, BUN, UA, eGFR, and ACR. Survival curves were plotted using the Kaplan–Meier method, and the log-rank test was used to compare survival differences between groups. Subgroup analyses were conducted to evaluate the consistency of the treatment effect across different clinical characteristics. A *p* value < 0.05 was considered statistically significant. Statistical analyses were conducted using R statistical software version 4.5.0 (R Foundation for Statistical Computing, Vienna, Austria). Due to the imbalance in sample size between the CHPF group and the control group, PSM was performed using a 1:1 nearest-neighbor matching algorithm with a caliper width of 0.02 to minimize potential confounding factors. The key covariates included in the PSM model were as follows: sex, age, diabetes mellitus, hypertension, coronary heart disease, use of nephrotoxic agents, AKI stage, ALB, eGFR, UA, and ACR.

## 3. Results

### 3.1. Baseline characteristics

A total of 273 patients were initially screened. After applying the inclusion and exclusion criteria, 205 patients were finally enrolled. The median age of the cohort was 67.00 years (IQR: 59.00–72.00), with males accounting for 65.9% and females for 34.1%. Comparisons revealed no significant differences in most baseline characteristics between the two groups; however, the prevalence of coronary heart disease was significantly higher in the CHPF group than in the control group (*p* < 0.05) (Table 1).

**Table 1.** Baseline characteristics of the study population.

Characteristics	Total (*n* = 205)	CHPF group (*n* = 115)	Control group (*n* = 90)	*p* Value
Age (years)	67.00 (59.00, 72.00)	67.00 (59.00, 72.00)	66.50 (57.25, 72.00)	0.758
Sex, *n* (%)				0.301
Male	135 (65.9)	72 (62.6)	63 (70.0)	
Female	70 (34.1)	43 (37.4)	27 (30.0)	
BMI, kg/m^2^	23.43 ± 3.08	23.70 ± 3.19	23.08 ± 2.92	0.151
SBP, mmHg	125.61 ± 19.01	126.58 ± 19.83	124.37 ± 17.93	0.409
DBP, mmHg	72.14 ± 14.94	73.50 ± 16.07	70.40 ± 13.25	0.141
Diabetes mellitus, *n* (%)				1.000
No	120 (58.5)	67 (58.3)	53 (58.9)	
Yes	85 (41.5)	48 (41.7)	37 (41.1)	
Hypertension, *n* (%)				0.858
No	39 (19.0)	21 (18.3)	18 (20.0)	
Yes	166 (81.0)	94 (81.7)	72 (80.0)	
Coronary heart disease, *n* (%)				0.038
No	163 (79.5)	85 (73.9)	78 (86.7)	
Yes	42 (20.5)	30 (26.1)	12 (13.3)	
RAASi use, *n* (%)				0.882
No	155 (75.6)	86 (74.8)	69 (76.7)	
Yes	50 (24.4)	29 (25.2)	21 (23.3)	
Statin use, *n* (%)				1.000
No	174 (84.9)	98 (85.2)	76 (84.4)	
Yes	31 (15.1)	17 (14.8)	14 (15.6)	
AKI stage, *n* (%)				0.287
Stage 1	166 (81.0)	90 (78.3)	76 (84.4)	
Stage 2	39 (19.0)	25 (21.7)	14 (15.6)	
AKI etiology, *n* (%)				0.372
Nephrotoxic agent	25 (12.2)	12 (10.4)	13 (14.4)	
Infection	28 (13.7)	16 (13.9)	12 (13.3)	
Other causes	35 (17.1)	24 (20.9)	11 (12.2)	
Unclear	117 (57.1)	63 (54.8)	54 (60.0)	
ALB, g/L	36.01 ± 6.48	36.17 ± 6.37	35.80 ± 6.64	0.687
SCr, μmol/L	274.67 ± 139.43	263.89 ± 124.36	288.45 ± 156.23	0.224
BUN, mmol/L	15.55 ± 7.46	15.39 ± 7.41	15.74 ± 7.56	0.747
UA, μmol/L	464.43 ± 133.16	463.02 ± 117.41	466.23 ± 151.60	0.869
eGFR, mL/min/1.73 m^2^	27.09 ± 15.60	27.14 ± 15.28	27.03 ± 16.08	0.962
ACR, mg/g	311.10 (36.10, 1,226.00)	311.10 (30.10, 1,598.10)	309.80 (38.30, 1,127.58)	0.864

BMI: body mass index; SBP: systolic blood pressure; DBP: diastolic blood pressure; RAASi: renin–angiotensin–aldosterone system inhibitors; AKI: acute kidney injury; ALB: albumin; SCr: serum creatinine; BUN: blood urea nitrogen; UA: uric acid; eGFR: estimated glomerular filtration rate; ACR: albumin-to-creatinine ratio.

To minimize selection bias and potential confounding factors, PSM was performed. After matching, 81 patients were retained in each group. All baseline characteristics were well-balanced between the two groups, with no statistically significant differences observed (Table 2).

**Table 2.** Baseline characteristics of the study population after propensity score matching.

Characteristics	Total (*n* = 162)	CHPF group (*n* = 81)	Control group (*n* = 81)	*p* Value
Age, (years)	66.00 (58.00, 72.00)	66.00 (59.00, 71.00)	66.00 (58.00, 72.00)	0.903
Sex, *n* (%)				0.620
Male	106 (65.4)	51 (63.0)	55 (67.9)	
Female	56 (34.6)	30 (37.0)	26 (32.1)	
BMI, kg/m^2^	23.18 ± 2.93	23.33 ± 2.94	23.03 ± 2.94	0.509
SBP, mmHg	124.81 ± 19.43	125.91 ± 20.72	123.70 ± 18.11	0.471
DBP, mmHg	70.85 ± 14.55	71.59 ± 15.70	70.10 ± 13.36	0.515
Diabetes mellitus, *n* (%)				0.872
No	100 (61.7)	51 (63.0)	49 (60.5)	
Yes	62 (38.3)	30 (37.0)	32 (39.5)	
Hypertension, *n* (%)				1.000
No	37 (22.8)	19 (23.5)	18 (22.2)	
Yes	125 (77.2)	62 (76.5)	63 (77.8)	
Coronary heart disease, *n* (%)				1.000
No	138 (85.2)	69 (85.2)	69 (85.2)	
Yes	24 (14.8)	12 (14.8)	12 (14.8)	
RAASi use, *n* (%)				0.726
No	117 (72.2)	57 (70.4)	60 (74.1)	
Yes	45 (27.8)	24 (29.6)	21 (25.9)	
Statin use, *n* (%)				1.000
No	137 (84.6)	68 (84.0)	69 (85.2)	
Yes	25 (15.4)	13 (16.0)	12 (14.8)	
AKI stage, *n* (%)				1.000
Stage 1	133 (82.1)	66 (81.5)	67 (82.7)	
Stage 2	29 (17.9)	15 (18.5)	14 (17.3)	
AKI etiology, *n* (%)	0.277			
Nephrotoxic agent	20 (12.3)	11 (13.6)	9 (11.1)	
Infection	23 (14.2)	12 (14.8)	11 (13.6)	
Other causes	28 (17.3)	18 (22.2)	10 (12.3)	
Unclear	91 (56.2)	40 (49.4)	51 (63.0)	
ALB, g/L	36.31 ± 6.50	36.25 ± 6.81	36.38 ± 6.22	0.894
SCr, μmol/L	267.01 ± 142.32	248.94 ± 122.83	285.07 ± 158.16	0.107
BUN, mmol/L	15.25 ± 7.61	15.17 ± 7.98	15.34 ± 7.27	0.882
UA, μmol/L	459.73 ± 131.09	459.24 ± 112.66	460.23 ± 147.96	0.962
eGFR, mL/min/1.73 m^2^	28.41 ± 16.07	29.23 ± 15.79	27.58 ± 16.41	0.515
ACR, mg/g	279.15 (36.35, 1,111.33)	205.60 (30.40, 1,108.70)	299.00 (37.10, 1,112.20)	0.809

BMI: body mass index; SBP: systolic blood pressure; DBP: diastolic blood pressure; RAASi: renin–angiotensin–aldosterone system inhibitors; AKI: acute kidney injury; ALB: albumin; SCr: serum creatinine; BUN: blood urea nitrogen; UA: uric acid; eGFR: estimated glomerular filtration rate; ACR: albumin-to-creatinine ratio.

### 3.2. Primary outcomes

**Figure 1.**
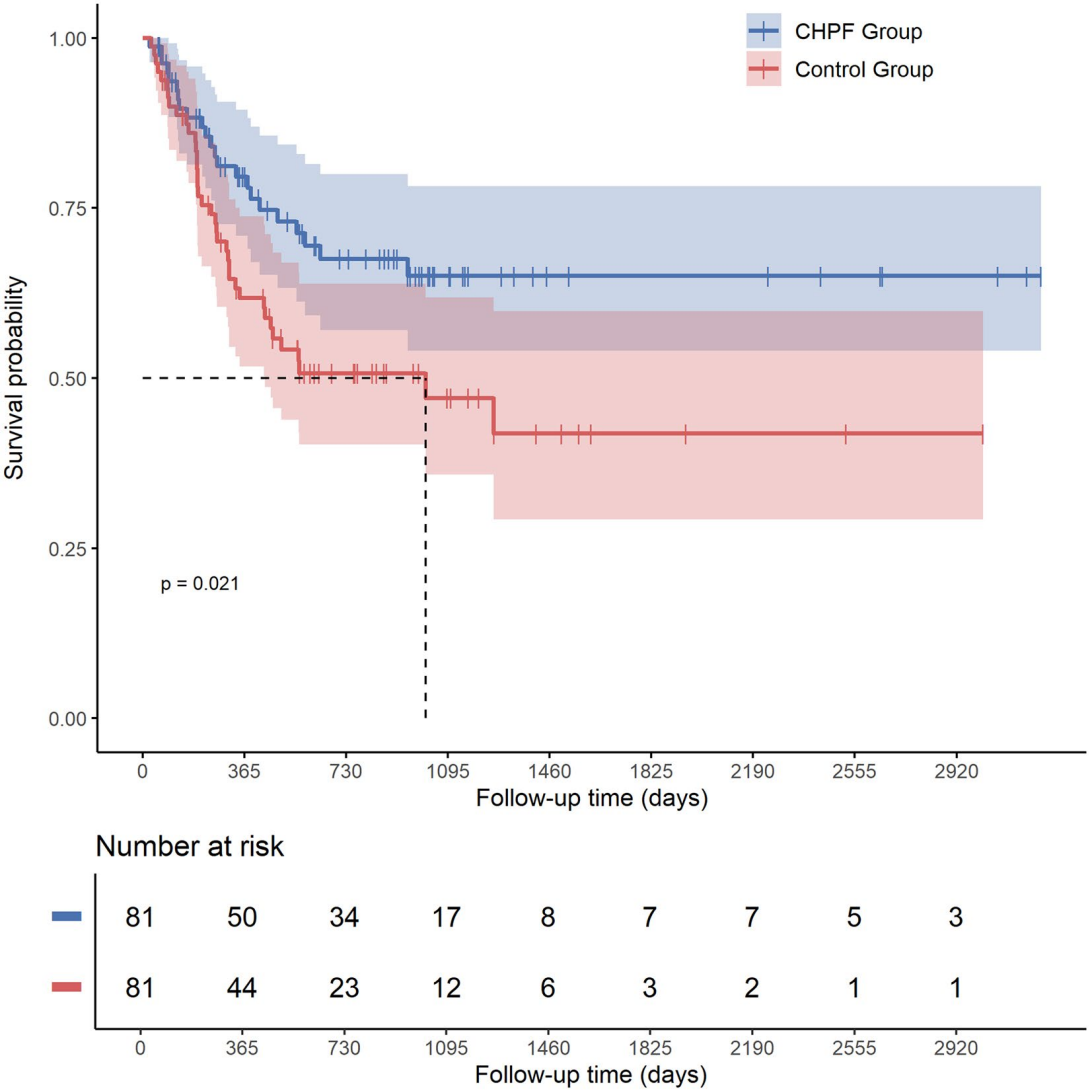
Kaplan–Meier’s survival curve analysis for the two groups after propensity score matching. Survival differences between groups were compared using the log-rank test.

Kaplan–Meier’s analysis showed that the median time to the primary outcome in the control group was 1,015 days [95% confidence interval (CI): 440–not reached], whereas it was not reached in the CHPF group during the follow-up period. The outcome-free survival rate in the CHPF group was significantly higher than that in the control group (log-rank *p* = 0.021) (Figure 1). The early steep decline of the Kaplan–Meier curves might be partly explained by the limited renal reserve of patients with A on C, as some patients could rapidly progress to adverse renal outcomes shortly after the AKI episode.

**Figure 2.**
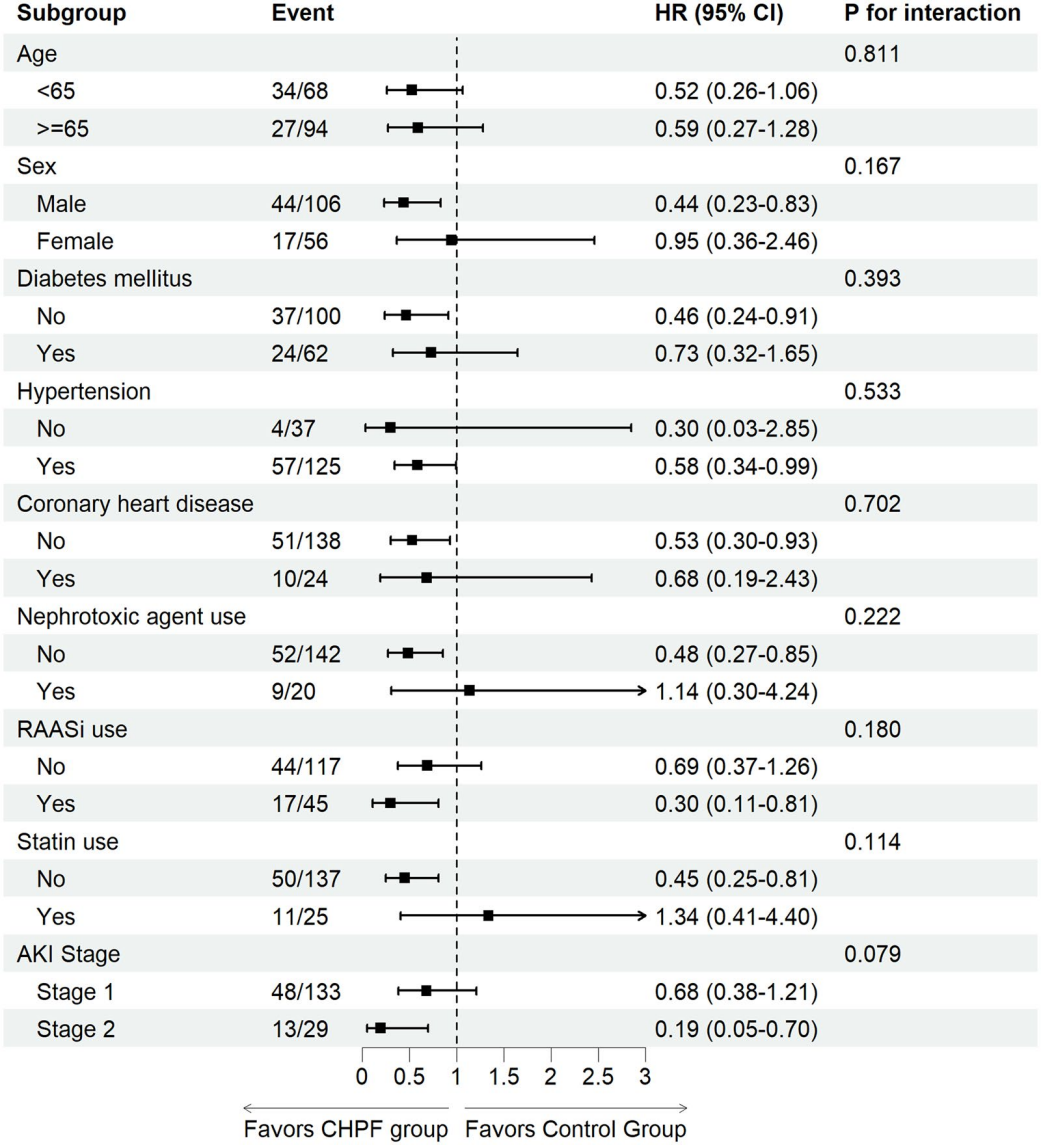
Subgroup analysis for CHPF group versus control group. Hazard ratios and 95% confidence intervals were estimated using Cox proportional hazards regression models, and *p* values for interaction were calculated using interaction terms.

To further quantify this association and adjust for potential confounders, Cox proportional hazards regression analyses were performed (Table 3). In the unadjusted model (model 1), CHPF treatment was associated with a lower risk of the primary outcome (hazard ratio (HR) 0.55, 95% CI 0.33–0.92, *p* = 0.023). This association remained consistent in the partially adjusted model (model 2: HR 0.54, 95% CI 0.32–0.91, *p* = 0.021). In the fully adjusted model (model 3), CHPF treatment remained associated with a 44% lower risk of the primary outcome compared with the control group (HR 0.56, 95% CI 0.32–0.98, *p* = 0.041). Subgroup analyses suggested that the association between CHPF treatment and the primary outcome was generally consistent across prespecified subgroups (Figure 2).

**Table 3.** Cox proportional hazards models for the primary composite outcome, defined as progression to CKD stage 5, initiation of maintenance RRT, or all-cause mortality.

	Model 1		Model 2		Model 3	
Group	HR (95% CI)	*p* Value	HR (95% CI)	*p* Value	HR (95% CI)	*p* Value
Control group	Ref.		Ref.		Ref.	
CHPF group	0.55 (0.33–0.92)	0.023	0.54 (0.32–0.91)	0.021	0.56 (0.32–0.98)	0.041

CKD: chronic kidney disease; RRT: renal replacement therapy; HR: hazard ratio; CI: confidence interval; BMI: body mass index; SBP: systolic blood pressure; DBP: diastolic blood pressure; RAASi: renin–angiotensin–aldosterone system inhibitors; AKI: acute kidney injury; ALB: albumin; BUN: blood urea nitrogen; UA: uric acid; eGFR: estimated glomerular filtration rate; ACR: albumin-to-creatinine ratio.

Model 1: unadjusted. Model 2: adjusted for age and sex. Model 3: adjusted for age, sex, BMI, SBP, DBP, diabetes mellitus, hypertension, coronary heart disease, RAASi use, statin use, AKI stage, AKI etiology, ALB, BUN, UA, eGFR, and ACR.

### 3.3. Secondary outcomes

**Figure 3.**
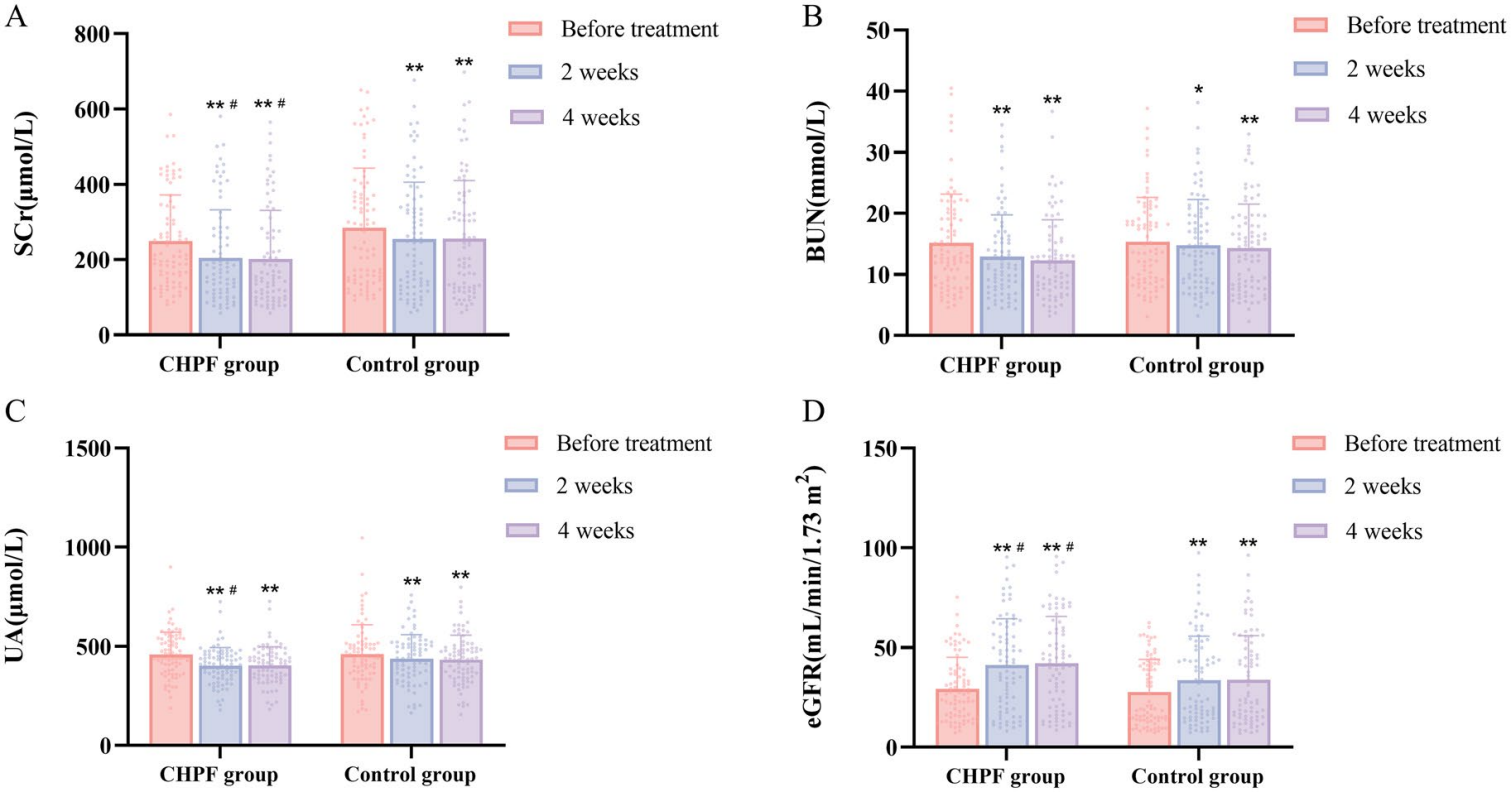
Comparison of secondary outcomes between the two groups before treatment and at 2 and 4 weeks after treatment initiation. (A) Serum creatinine (SCr); (B) blood urea nitrogen (BUN); (C) uric acid (UA); and (D) estimated glomerular filtration rate (eGFR). Intragroup comparisons were performed using paired *t*-tests or Wilcoxon’s signed-rank tests, and intergroup comparisons were performed using independent samples *t*-tests or Mann–Whitney’s *U*-tests, as appropriate. Significance is indicated as the *p* value. *p < 0.05, **p < 0.01, vs. before treatment. ^#^*p* < 0.05, vs. control group.

To evaluate short-term and subsequent changes in renal function parameters associated with CHPF treatment, we compared SCr, BUN, UA, and eGFR between the two groups before treatment and at 2 and 4 weeks after treatment initiation (Table 4; Figure 3). Before treatment, there were no significant differences in baseline levels of SCr, BUN, UA, and eGFR between the CHPF group and the control group (*p* > 0.05). At 2 weeks after treatment, renal function parameters showed favorable changes in both groups compared with before treatment. In the intergroup comparison, the CHPF group had significantly lower SCr and UA levels and a significantly higher eGFR than the control group (all *p* < 0.05). At 4 weeks after treatment, the CHPF group continued to show significantly lower SCr levels and significantly higher eGFR compared with the control group (both *p* < 0.05). BUN and UA levels also showed downward trends at 4 weeks, although no statistically significant between-group differences were observed.

**Table 4.** Comparison of secondary outcomes, defined as renal function parameters at 2 and 4 weeks after treatment, between two groups.

	CHPF group (*n* = 81)			Control group (*n* = 81)		
Outcomes	Before treatment	2 weeks	4 weeks	Before treatment	2 weeks	4 weeks
SCr, μmol/L	248.94 ± 122.83	204.61 ± 127.87**^,#^	201.93 ± 128.98**^,#^	285.07 ± 158.16	254.78 ± 151.21**	255.80 ± 154.11**
BUN, mmol/L	15.17 ± 7.98	12.92 ± 6.84**	12.30 ± 6.66**	15.34 ± 7.27	14.75 ± 7.50*	14.30 ± 7.24**
UA, μmol/L	459.24 ± 112.66	401.26 ± 92.12**^,#^	402.50 ± 94.38**	460.23 ± 147.96	436.21 ± 122.16**	432.50 ± 123.19**
eGFR, mL/min/1.73 m^2^	29.23 ± 15.79	41.19 ± 23.26**^,#^	42.05 ± 23.51**^,#^	27.58 ± 16.41	33.58 ± 22.14**	33.67 ± 22.30**

SCr: serum creatinine; BUN: blood urea nitrogen; UA: uric acid; eGFR: estimated glomerular filtration rate.

Significance is indicated as the *p* value.

*p < 0.05, **p < 0.01, vs. before treatment.

^#^*p* < 0.05, vs. control group.

### 3.4. Kaplan–Meier’s survival analysis stratified by hypertension

**Figure 4.**
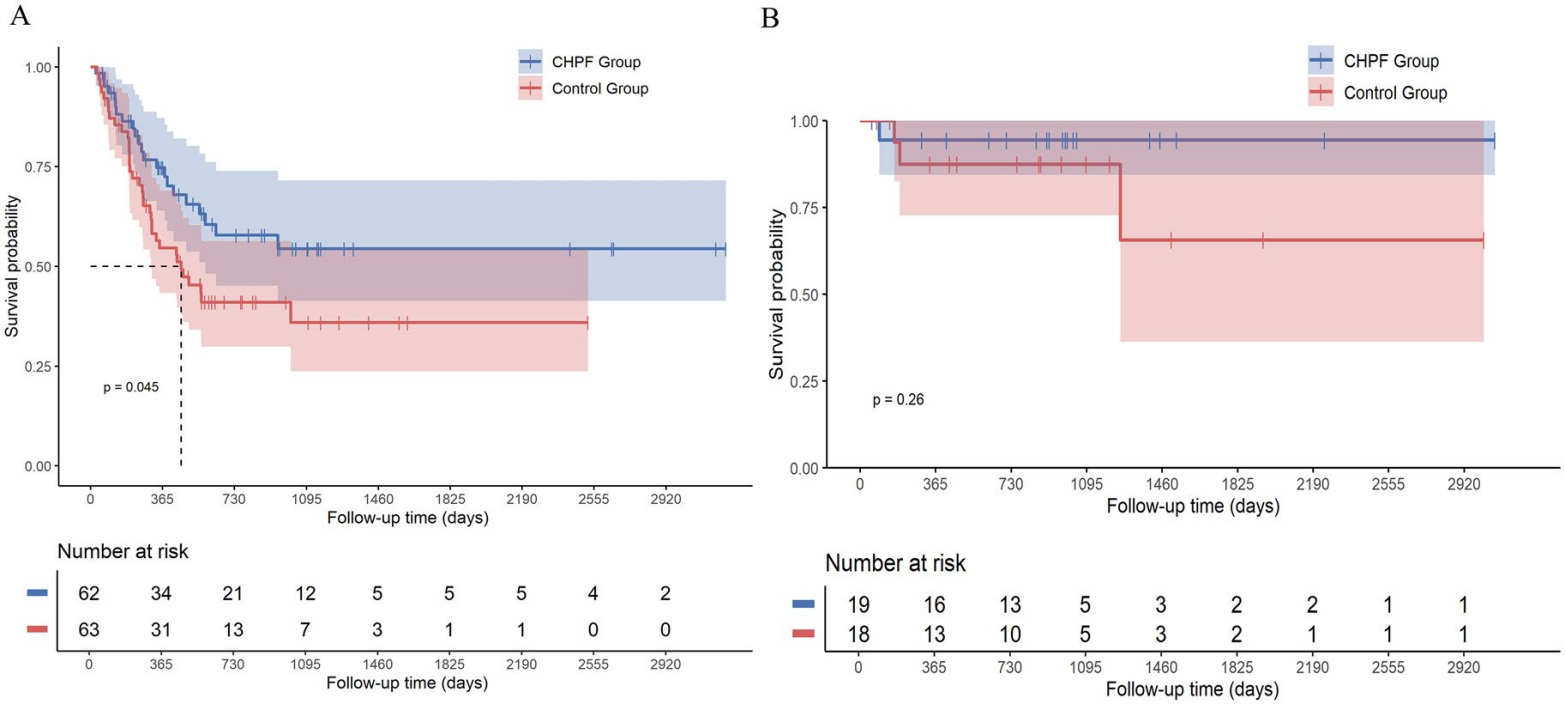
Kaplan–Meier’s survival curve analysis for the two groups stratified by hypertension. (A) Kaplan–Meier’s survival curves for patients with hypertension. (B) Kaplan–Meier’s survival curves for patients without hypertension. Survival differences between groups were compared using the log-rank test.

Given the strong association between hypertension and renal prognosis, we further stratified the survival analysis based on whether patients had comorbid hypertension. In patients with comorbid hypertension, the median time to the primary outcome in the control group was 460 days (95% CI: 310–not reached), whereas it was not reached in the CHPF group during the follow-up period. Kaplan–Meier’s curves indicated that in this subgroup, the outcome-free survival rate in the CHPF group was significantly higher than that in the control group (log-rank *p* = 0.045; Figure 4(A)). Conversely, in patients without comorbid hypertension, the median time to the primary outcome was not reached in either the control group or the CHPF group. In this subgroup, no significant difference in outcome-free survival was observed between the two groups (log-rank *p* = 0.26; Figure 4(B)). These findings suggest that the association between CHPF treatment and outcome-free survival might be more evident in patients with comorbid hypertension; however, this subgroup finding should be interpreted cautiously because the interaction test was not statistically significant.

## 4. Discussion

This retrospective cohort study provides real-world clinical evidence regarding the use of CHPF in patients with A on C. Most available clinical evidence regarding TCM-based interventions in kidney diseases has focused on short-term biochemical indicators or symptomatic outcomes, whereas data on long-term clinically meaningful renal outcomes in patients with A on C remain limited. Our results indicate that CHPF treatment was associated with more favorable long-term clinical prognosis. After adjusting for multiple confounding factors, CHPF treatment was associated with a 44% lower risk of the primary outcome (progression to CKD stage 5, initiation of dialysis, or all-cause mortality). Additionally, regarding short-term efficacy, our results showed that renal function parameters at 2 and 4 weeks after treatment initiation were generally more favorable in the CHPF group than in the control group. Notably, subgroup analysis stratified by comorbid hypertension suggested that the association between CHPF treatment and long-term prognosis appeared more evident in patients with comorbid hypertension. However, because the interaction test was not statistically significant, this finding should be interpreted as exploratory rather than as evidence of a definitive differential treatment effect.

Common triggers for A on C include infection, electrolyte disturbances, hypertension, and stress states [23]. There is a bidirectional relationship between AKI and CKD: patients with AKI are prone to progressing to CKD, while those with preexisting CKD are more susceptible to risk factors for AKI, with repeated injuries accelerating the deterioration of renal function [31]. Currently, various pathophysiological or metabolic processes have been proven to participate in AKI or the transition from AKI to CKD, such as renal tubular epithelial cell injury or maladaptive repair, immune-mediated inflammation, and microcirculatory disturbances [32–34]. A recent study further showed that WNT10B/FOXO6 signaling could promote renal tubular cell fate transition, cellular senescence, inflammation, and fibrogenesis, providing new mechanistic evidence for the role of tubular maladaptive repair in kidney disease progression [35]. However, current clinical practice faces severe challenges. Although multiple studies have indicated that AKI is a risk factor for the occurrence and progression of CKD [36–38], in clinical settings, this pathological process of transition from acute injury to chronic fibrosis is often insidious and rapid. Once this pathway is not timely interrupted, RRT alone is often difficult to reverse the continuous loss of renal function, leading to a faster progression to ESRD.

Preexisting CKD also increases susceptibility to AKI. On the background of chronic pathology, the renal tolerance threshold to ischemia or toxins is lowered, leading to more severe tissue destruction upon acute insults. Concurrently, the endogenous regenerative capacity of the CKD kidney is significantly impaired, impeding functional recovery through normal adaptive repair mechanisms, which often results in a poor prognosis for A on C patients [39]. Therefore, the A on C stage represents a critical period in the course of CKD; timely disease control could lead to marked improvements in long-term prognosis. Our study results suggest that CHPF administration after AKI onset in patients with A on C was associated with more favorable short-term renal function parameters at 2 and 4 weeks and a lower risk of long-term adverse outcomes, including progression to CKD stage 5, initiation of maintenance RRT, and all-cause mortality.

A notable finding of this study concerns the subgroup analysis of patients with comorbid hypertension. We observed that the association between CHPF treatment and long-term outcome-free survival appeared more evident in this population. A close causal relationship exists between hypertension and the onset and progression of CKD; hypertension serves not only as a complication of CKD but also as a critical factor exacerbating renal ischemia [40,41]. In CKD patients, renal parenchymal damage frequently leads to aberrant activation of the RAAS and sodium-water retention, thereby inducing hypertension. Conversely, a sustained hypertensive state triggers renal arteriolosclerosis and hyalinosis, resulting in elevated renal vascular resistance and impaired perfusion, thus forming a vicious cycle [42,43]. Under hypertensive conditions, activation of the sympathetic nervous system causes renal vasoconstriction and ischemia, which subsequently induces podocyte injury, glomerulosclerosis, and interstitial fibrosis, driving the continuous decline of renal function [44]. Tetramethylpyrazine (TMP), an alkaloid compound isolated from the Chinese herb Chuanxiong, has been verified to possess properties such as expanding renal vessels, improving microcirculation, inhibiting platelet aggregation, and exerting anti-inflammatory and anti-fibrotic effects [45,46]. TMP is capable of counteracting hypertension-induced hemodynamic disturbances, thereby alleviating renal ischemic injury by ameliorating renal microcirculatory perfusion. Our previous research indicated that TMP exerts renoprotective effects in contrast-induced nephropathy by inhibiting TFRC and intracellular ROS production, thereby attenuating ferroptosis [47]. Furthermore, we demonstrated that TMP might alleviate arsenite-induced AKI by ameliorating autophagic flux blockade via the YAP1–Nrf2–p62 pathway [48]. Furthermore, the CHPF group had lower post-treatment serum UA levels than the control group, and hyperuricemia is closely associated with hypertension [49]. Hyperuricemia acts as a pivotal factor exacerbating hypertension and vascular pathology; its mechanisms involve the activation of the RAAS and the reduction of endothelial nitric oxide bioavailability via oxidative stress induction, leading to sustained vasoconstriction and endothelial dysfunction [50]. Therefore, CHPF might be related to the modulation of the vicious cycle between hypertension and renal injury through potential mechanisms involving microcirculatory improvement and metabolic regulation, which might partly explain the more evident association observed in patients with comorbid hypertension. Although the interaction test did not reach statistical significance (*p* = 0.533), possibly due to sample size limitations, the log-rank result observed in patients with comorbid hypertension (*p* = 0.045) suggests that this subgroup could warrant further investigation. Therefore, this finding should be interpreted cautiously and regarded as exploratory.

However, several limitations of this study must be acknowledged. First, as a retrospective observational study, inherent biases are unavoidable. Although we adjusted for multiple confounding factors using statistical methods, the influence of unmeasured variables on the results cannot be entirely ruled out. Among the measured baseline variables, SCr was not included in the Cox models to avoid collinearity with eGFR. Second, given that this was a single-center study conducted in a TCM hospital and that comorbid hypertension was relatively common in the study population, the generalizability of these findings to broader CKD populations or international settings might be limited. Finally, the limited sample size restricted the statistical power for interaction tests in subgroup analyses; therefore, the findings regarding the potential benefit in patients with comorbid hypertension should be interpreted as exploratory and require confirmation in larger populations.

## 5. Conclusions

In conclusion, CHPF treatment was associated with a lower long-term risk of the primary composite outcome – including progression to CKD stage 5, initiation of maintenance RRT, and all-cause mortality – in patients with A on C. Additionally, CHPF treatment was associated with more favorable short-term renal function parameters at 2 and 4 weeks. Patients with comorbid hypertension might represent a potential subgroup that could derive greater benefit from CHPF treatment; however, this exploratory finding should be interpreted cautiously because the interaction test was not statistically significant. These findings suggest that CHPF might be a promising adjunctive strategy for the treatment of A on C.

## Data Availability

The data in this study are available from the corresponding author on reasonable request.
